# Supplementary material for: Observation of magnon-mediated current drag in Pt/yttrium iron garnet/Pt(Ta) trilayers
Source: Nat Commun. 2016 Mar 2;7:10858. doi: 10.1038/ncomms10858 (PMC4778061; doi:10.1038/ncomms10858)
Supplement: Supplementary Information — Supplementary Figures 1-6, Supplementary Notes 1-5 and Supplementary References [file ncomms10858-s1.pdf]

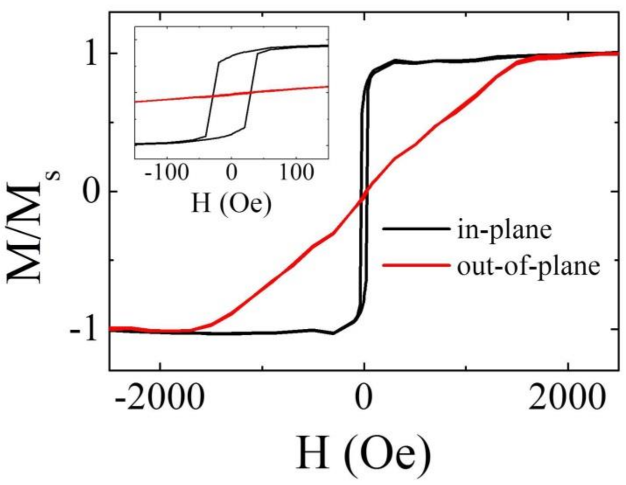

Supplementary Figure 1. Magnetic properties of YIG film. Room temperature magnetic hysteresis loops of an unpatterned GGG/Pt(5 nm)/YIG(80 nm) film measured by vibrating sample magnetometry (VSM). Inset shows the zoom-in low-field hysteresis loops.

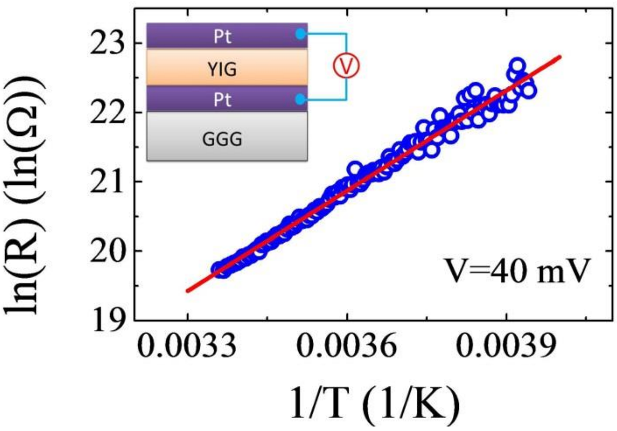

Supplementary Figure 2. Leakage test of the device. Resistance between the top and bottom Pt layers vs. 1/T for a fixed bias voltage of 40 mV as shown in the inset. The thickness of YIG is 80 nm. The temperature range is from 300 to 250 K, in which the corresponding resistance increases from 0.3 to 6 GΩ. The red line is a fit which gives an effective barrier height of 0.42 eV over this temperature range. Below this temperature range, the resistance keeps increasing exponentially, but the voltage has to be adjusted to higher values, which results in a slightly different barrier height due to some bias voltage dependence.

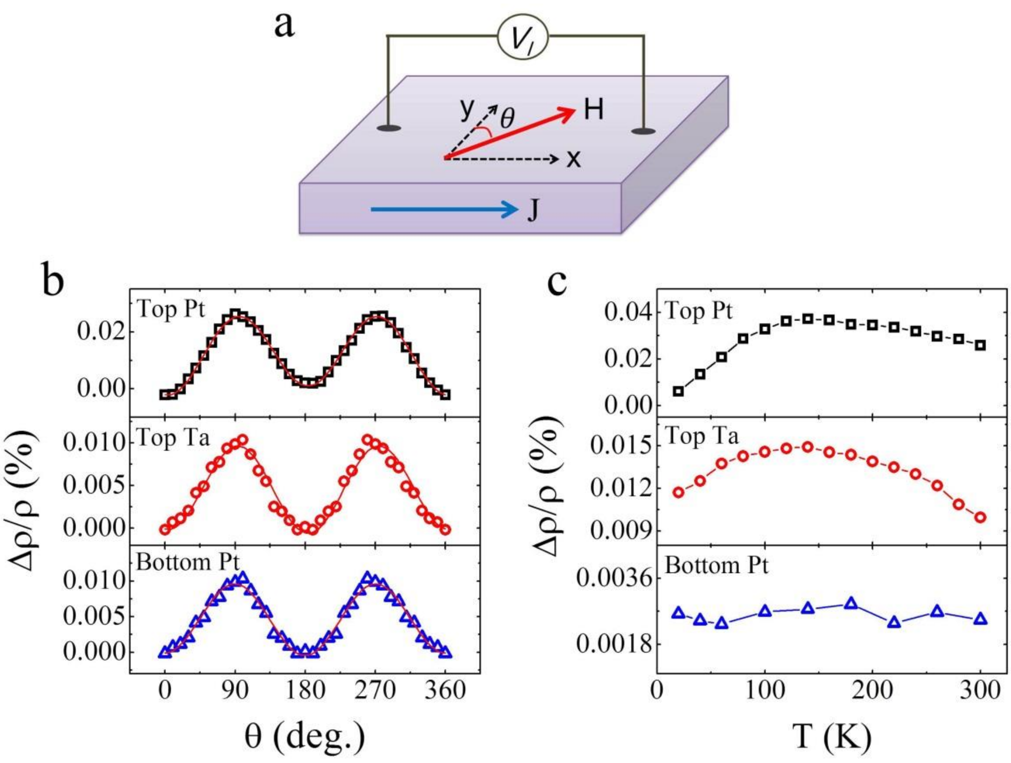

Supplementary Figure 3. Spin Hall magnetoresistance of normal metal. a, Schematic illustration of local MR measurement geometry. A magnetic field is applied in the film plane at an angle θ with the y-axis. **b,** Room-temperature angular dependence of the MR in the top Pt, top Ta, and bottom Pt layers with a magnetic field of 1000 Oe. Continuous red curves are the fitting results using Supplementary equation (1). **c,** Temperature dependence of the MR ratio for the top and bottom normal metals.

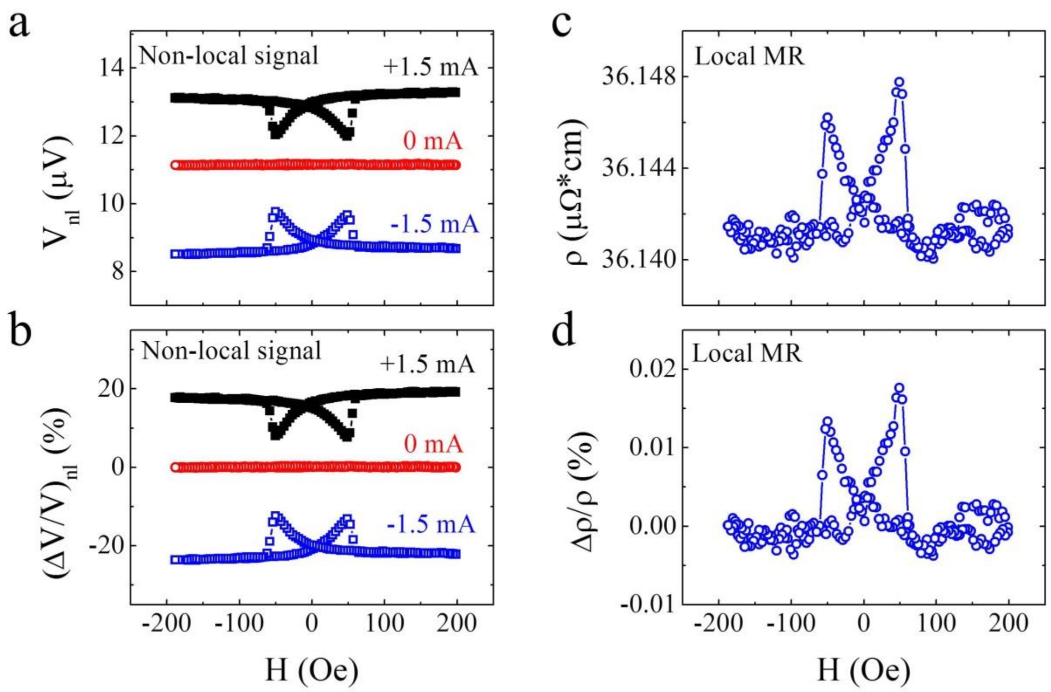

Supplementary Figure 4. Field-dependent nonlocal and local signals. a and **b** are raw nonlocal data and the relative change of the nonlocal signal with respect to V_{nl} (0 mA), respectively, in Pt/YIG/Pt device at 220 K; black, red, and blue symbols represents V_{nl} at +1.5 mA, 0 mA, and -1.5 mA of the bottom layer current. **c** and **d** are the local resistivity and MR ratio for the top Pt layer in Pt/YIG/Pt device at 220 K.

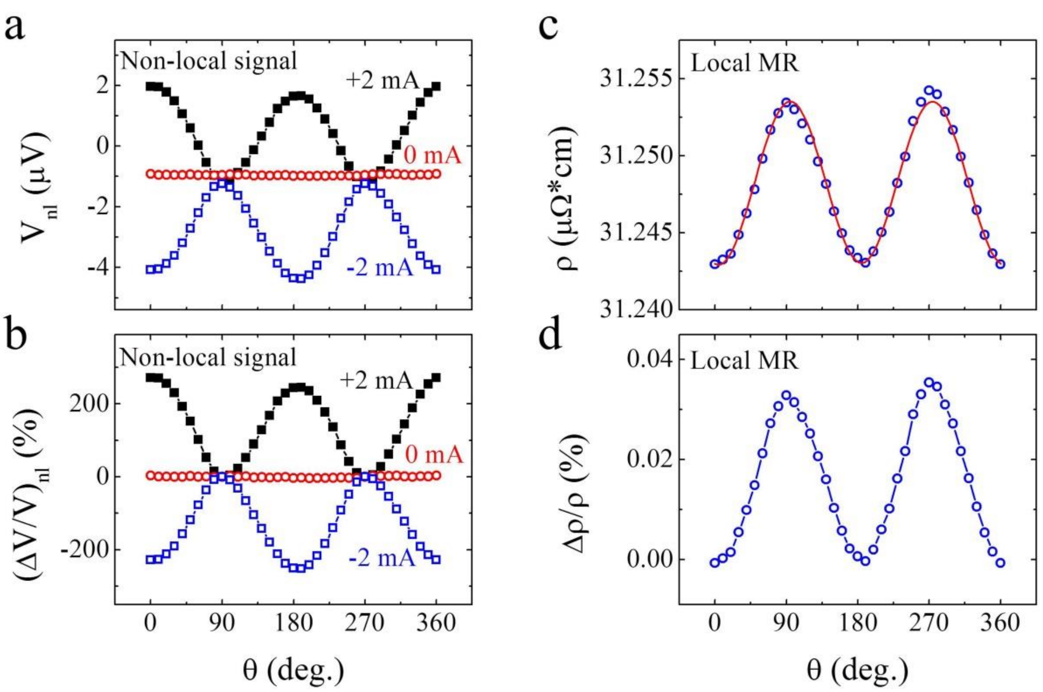

Supplementary Figure 5. Angular dependent nonlocal and local signals. a and **b** are the raw nonlocal signal and the relative change of the nonlocal signal with respect to V_{nl} (0 mA), respectively, in Pt/YIG/Pt device at 220 K; black, red and blue symbols represent V_{nl} at +2 mA, 0 mA, and -2 mA in the bottom current. **c** and **d** are the local resistivity and the MR ratio for the top Pt layer in Pt/YIG/Pt device at 220 K. The red solid curve in **c** is the results from fitting the Supplementary Equation (1).

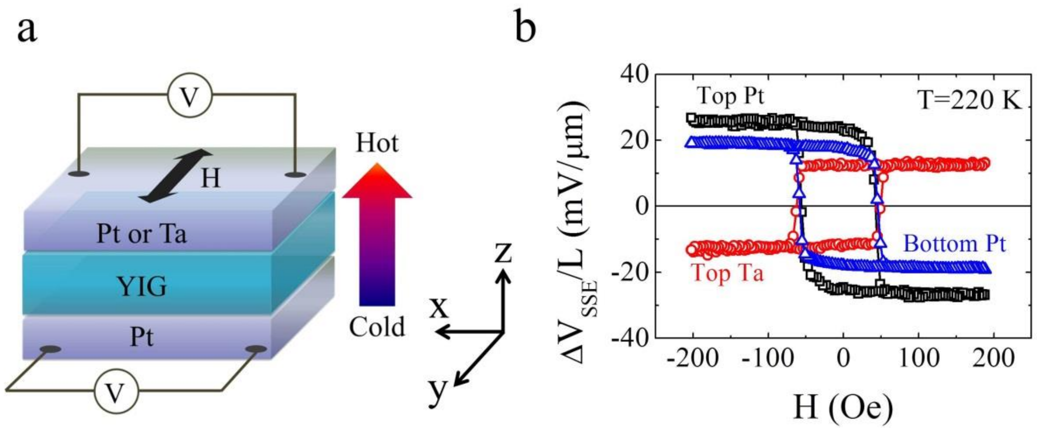

Supplementary Figure 6. Longitudinal spin Seebeck effect in sandwich devices. **a,** Schematic illustration of the longitudinal spin Seebeck effect measurement set-up. A temperature gradient was generated along the z-direction by a heater on top. Voltages of the top layer and bottom layers were measured along the x-direction with the magnetic field sweeping in the y-direction. **b,** SSE signals divided by the detecting stripe length L of the top Pt, top Ta, and bottom Pt layers as a function of the magnetic field at T=220 K.

Supplementary Note 1. Magnetic properties of YIG film deposited on Pt

The magnetic properties of YIG grown on GGG by pulsed laser deposition have been well studied in previous works[1-4]. In this work, YIG was deposited on 5 nm thick Pt. After post-annealing at 800 ^{o}C for 200 s, the hysteresis was measured using a vibrating sample magnetometer (VSM). YIG is magnetized and has in-plane magnetic anisotropy, as shown in Supplementary Figure 1.

Supplementary Note 2. Leakage test of the sandwich device.

In sandwich structures, we measured the resistance between top and bottom normal metals (NMs). As illustrated in the inset of Supplementary Figure 2, we measured the resistance by applying a voltage between the top and bottom NMs and detecting the leakage current as a function of temperature. The bias voltage applied has to be adjusted in different temperature ranges because of the dramatic resistance change. The junction resistance increases rapidly with decreasing temperature and exceeds 20 $G\Omega$ for $\mathrm{T} \leq 220K$, which is larger than the input impedance of the nano-voltmeter (> 10 $G\Omega$ for Keithley 2182A), indicating that the shunting current in leakage produces a negligible voltage change in the top NM layer via the magnetoresistance during the nonlocal measurements. In Supplementary Figure 2, we show the resistance change vs. 1/T on a semi-log plot for a constant bias voltage. It clearly shows an activated behavior with an effective barrier height of 0.42 eV for this temperature range. For different bias voltages, the extracted barrier height varies. This effective barrier height is much smaller than the band gap of YIG. We know that the YIG film is uniform in thickness and atomically flat. In addition, the film is insulating laterally. We believe that the observed activated behavior could be due to microscopic regions with relatively lower energy barriers. As the temperature is lowered, the whole film becomes highly insulating.

Supplementary Note 3. Spin Hall magnetoresistance of normal metal layers.

Spin Hall magnetoresistance is a unique transport phenomenon in the normal

metal which is in direct contact with a magnetic insulator[1,2,5-9]. A charge current flowing in the normal metal with strong spin-orbit coupling is converted to a spin current via the spin Hall effect. The reflection and absorption of this spin current at the interface of the normal metal/magnetic insulator depends on the orientation of the magnetization (**M**) of the magnetic insulator. When **M** is collinear to the spin polarization $\boldsymbol{\sigma}$, most of the spin current is reflected back; in contrast, when **M** is perpendicular to the spin polarization of the spin current ($\boldsymbol{M} \perp \boldsymbol{\sigma}$), most of the spin current is absorbed by the insulator. This behaves as a dissipation channel; therefore, the resistance of the normal metal is larger than that for $\boldsymbol{M} \parallel \boldsymbol{\sigma}$. As a result, the degree of refection or absorption of the spin current at the normal metal/magnetic insulator interface can be monitored by SMR. In order to characterize the quality of the interface in the sandwich device, we performed the MR measurements.

As illustrated in Supplementary Figure 3a, we carried out local MR measurements by applying a magnetic field (1000 Oe) in the film plane at an angle with the y-axis, while the current flows along the x-axis. The angular dependence of the MR ratios for top Pt, top Ta, and bottom Pt at room temperature are summarized in Supplementary Figure 3b. According to the SMR theory[6], the longitudinal resistivity reads as

$$\rho = \rho_0 + \rho_1 m_y^2 \qquad (1)$$

where ρ_0 is a constant insensitive to the magnetization orientation, ρ_1 represents the SMR magnitude which depends on film thickness, spin Hall angle and spin diffusion length of the normal metal, and spin-mixing interface conductance; and m_y is the y component of the magnetization unit vector. From the red solid curves in Supplementary Figure 3b, we found that the experimental data can be well described by Supplementary Equation (1).

Besides the SMR, the induced magnetization in the NM due to the magnetic proximity effect (MPE)[10-12] can also generate similar MR. The MPE induced MR should increase monotonically with decreasing temperature[12], since both the induced

magnetic moment and the spin diffusion length increase at low temperatures. However, the SMR has a broad peak as a function of temperature[2]. To better understand the nature of the MR we observed and verify the SMR mechanism, we performed the temperature dependent measurements, and the results are summarized in Supplementary Figure 3c. Remarkably, for both top Pt and Ta, the MR ratio have a broad peak, as expected from the SMR theory, suggesting the SMR mechanism dominates the transport property of the top normal metal. Note however that the MR ratio of the bottom Pt is one order smaller than that of the top Pt and is relatively insensitive to temperature. Recently, Goennenwein et al.[13] reported a monotonically decreasing SMR signal with decreasing temperature. Those results suggest that the temperature dependence of SMR is very sensitive to the interface quality. The lack of clear temperature dependence of SMR in the bottom Pt here can be attributed to the less ideal quality of the bottom interface possibly due to oxidation occurred in rapid thermal annealing (RTA) at high temperatures. Based on the temperature dependence of the MR results, we demonstrate that the SMR mechanism dominates in our devices, and the anisotropic reflection and absorption of the spin current at the NM/YIG interfaces indicate excellent interface quality for spin current interconversion.

Supplementary Note 4. Nonlocal current drag signal versus local MR signal.

Here, we demonstrate that the nonlocal signal we observed in sandwich structures cannot be produced by a local SMR voltage of the top Pt due to the leakage current at the location of the top electrode. Supplementary Figures 4a and 4c show the field dependence of the raw nonlocal signal in the Pt/YIG/Pt sandwich structure and that of the resistivity in the top Pt layer, respectively. For the nonlocal signal, V_{nl} at zero bottom current is constant which defines a reference. V_{nl} at +1.5 mA (-1.5 mA) shows the clear a hysteresis behavior and attains the minimum (maximum) values around the coercivity field of YIG. The relative change of the nonlocal signal, $\left(\frac{V(\mathrm{I})-V(0)}{|V(0)|}\right)_{nl}=\left(\frac{\Delta V}{V}\right)_{nl}$, is shown in Supplementary Figure 4b, where $V(0)$ is

the nonlocal signal in the absence of the bottom current. In contrast, we show the MR ratio of the top Pt in the same device in Supplementary Figure 4d. Remarkably, the magnitude of $\left(\frac{\Delta V}{V}\right)_{nl}$ at 1.5 mA (~20%) is almost four orders of magnitude larger than that of the MR ratio in the top Pt layer (~0.02%). Similar conclusion can also be obtained from the angular dependence of both the nonlocal signal and local MR in the top Pt layer, as shown in Supplementary Figure 5. The $\left(\frac{\Delta V}{V}\right)_{nl}$ at 2.0 mA (~240%) is almost five order larger than MR ratio in the top Pt (~0.03%). The above comparison between the nonlocal voltage signal and the local MR signal in the top Pt strongly suggests that the nonlocal signal cannot be accounted for by the SMR effect in the top layer due to the leakage current.

Supplementary Note 5. Longitudinal spin Seebeck effect (SSE).

We confirmed the transmission of thermally excited spin currents through the NM/YIG interface by performing the longitudinal spin Seebeck effect in our sandwich structures. On top of the sandwich structures, we first deposited a 300 nm thick Al_2O_3 layer, used to electrically insulate the device, and the top heater layer consists of 5 nm Cr and 50 nm Au. When a charge current (30 mA) is applied to the Cr/Au layer, a temperature gradient is established in the z-direction by joule heating, as shown in Supplementary Figure 6a. During the SSE measurements, a magnetic field was applied in the y-direction while the voltage is detected along the x-direction. In Supplementary Figure 6b, we plot the field dependence of the longitudinal spin Seebeck signal at 220 K, which is normalized to the device length. First, all three metal layers show strong SSE signals. Second, we found that the magnitude of the SSE signal from the bottom Pt is on the same order as that from the top Pt layer, which is different from the contrast in SMR shown in Supplementary Figure 3c. The different behaviors of SMR and SSE indicate that the effect on the anisotropic reflection (or absorption) of a spin current due to the spin transfer torque is very

different from that on the transmission of the thermally excited spin current by the same NM/YIG interface. Third, we notice that the SSE signal from the top Ta shows the opposite sign to that from Pt, which can be accounted for by the fact that Pt and Ta have opposite spin Hall angles. Therefore, we have confirmed the excellence interface quality for transmitting thermally excited spin currents.

Supplementary References

1. Lin, T. *et al*. Induced magneto-transport properties at palladium/yttrium iron garnet interface. Appl. Phys. Lett. **103**, 132407 (2013).

2. Lin, T. *et al*. Experimental Investigation of the Nature of the Magnetoresistance Effects in Pd-YIG Hybrid Structures. Phys. Rev. Lett. **113**, 037103 (2014).

3. Jiang, Z. *et al*. A comparative transport study of Bi_2Se_3 and Bi_2Se_3/yttrium iron garnet. Appl. Phys. Lett. **104**, 222409 (2014).

4. Jiang Z. *et al*. Independent Tuning of Electronic Properties and Induced Ferromagnetism in Topological Insulators with Heterostructure Approach. *Nano Lett.* **15 (9)**, 5835–5840 (2015).

5. Nakayama, H. *et al*. Spin Hall Magnetoresistance Induced by a Non-equilibrium Proximity Effect. Phys. Rev. Lett. **110**, 206601 (2013).

6. Chen, Y.-T. *et al*. Theory of spin Hall magnetoresistance. Phys. Rev. B **87**, 144411 (2013).

7. Althammer, M. *et al*. Quantitative study of the spin Hall magnetoresistance in ferromagnetic insulator/normal metal hybrids. Phys. Rev. B **87**, 224401 (2013).

8. Hahn, C. *et al.* Comparative measurements of inverse spin Hall effects and magnetoresistance in YIG/Pt and YIG/Ta. Phys. Rev. B **87**, 174417 (2013).

9. Vlietstra, N. *et al.* Spin-Hall magnetoresistance in platinum on yttrium iron garnet: Dependence on platinum thickness and in-plane/out-of-plane magnetization. Phys. Rev. B **87**, 184421 (2013).

10. Huang, S. Y. *et al.* Transport magnetic proximity effects in platinum. Phys. Rev. Lett. **109**, 107204 (2012).

11. Lu, Y. M. *et al*. Hybrid magnetoresistance in the proximity of a ferromagnet. Phys. Rev. B **87**, 220409 (2013).

12. Lu, Y. M. *et al.* Pt Magnetic Polarization on $Y_3Fe_5O_{12}$ and Magnetotransport Characteristics. Phys. Rev. Lett. **110**, 147207 (2013).

13. Goennenwein, S. T. B., Schlitz, R., Pernpeintner, M., Ganzhorn, K., Althammer, M., Gross, R., and Huebl, H. Non-local magnetoresistance in YIG/Pt nanostructures. Appl. Phys. Lett. **107**, 172405 (2015).
